# Neuregulin 4 Downregulation Induces Insulin Resistance in 3T3-L1 Adipocytes through Inflammation and Autophagic Degradation of GLUT4 Vesicles

**DOI:** 10.3390/ijms222312960

**Published:** 2021-11-30

**Authors:** Francisco Díaz-Sáez, Carla Blanco-Sinfreu, Adrià Archilla-Ortega, David Sebastian, Montserrat Romero, Maria Isabel Hernández-Alvarez, Sílvia Mora, Xavier Testar, Wifredo Ricart, José Manuel Fernández-Real, José María Moreno-Navarrete, Julián Aragonés, Marta Camps, Antonio Zorzano, Anna Gumà

**Affiliations:** 1 Department of Biochemistry and Molecular Biomedicine, Faculty of Biology, University of Barcelona, Av. Diagonal, 643, 08028 Barcelona, Spain; frandiazsaez@gmail.com (F.D.-S.); carlablancosinfreu@outlook.com (C.B.-S.); adria.archilla.ortega@gmail.com (A.A.-O.); david.sebastian@irbbarcelona.org (D.S.); montserrat.romero@irbbarcelona.org (M.R.); mihernandez@ub.edu (M.I.H.-A.); smora@ub.edu (S.M.); xtestar@ub.edu (X.T.); martacamps@ub.edu (M.C.); 2 Institute of Biomedicine, University of Barcelona, 08028 Barcelona, Spain; 3 Centro de Investigación Biomèdica en Red Diabetes y Enfermedades Metabólicas Asociadas (CIBERDEM), Carlos III Health Institute, 28029 Madrid, Spain; 4 Institute for Research in Biomedicine (IRB Barcelona), Barcelona Institute of Science and Technology (BIST), 08028 Barcelona, Spain; 5 Department of Medicine, Universitat de Girona, Carrer Emili Grahit, 77, 17071 Girona, Spain; wricart@idibgi.org (W.R.); jmfreal@idibgi.org (J.M.F.-R.); jmoreno@idibgi.org (J.M.M.-N.); 6 Department of Diabetes, Endocrinology and Nutrition, Institut d’Investigació Biomèdica de Girona (IDIBGI), Carrer del Dr. Castany, s/n, 17190 Salt, Spain; 7 Centro de Investigación Biomèdica en Red Fisiopatología de la Obesidad y Nutrición (CIBEROBN), Carlos III Health Institute, 28029 Madrid, Spain; 8 Research Unit, Hospital of Santa Cristina, Research Institute Princesa, Autonomous University of Madrid, c/Maestro Vives, 2, 28009 Madrid, Spain; jaragones.hlpr@salud.madrid.org; 9 Centro de Investigación Biomédica en Red Enfermedades Cardiovasculares, Carlos III Health Institute, 28029 Madrid, Spain

**Keywords:** insulin resistance, neuregulin 4, adipocytes, autophagy, inflammation, insulin receptor, Glut4

## Abstract

The adipokine Neuregulin 4 (Nrg4) protects against obesity-induced insulin resistance. Here, we analyze how the downregulation of Nrg4 influences insulin action and the underlying mechanisms in adipocytes. Validated shRNA lentiviral vectors were used to generate scramble (Scr) and Nrg4 knockdown (KD) 3T3-L1 adipocytes. Adipogenesis was unaffected in Nrg4 KD adipocytes, but there was a complete impairment of the insulin-induced 2-deoxyglucose uptake, which was likely the result of reduced insulin receptor and Glut4 protein. Downregulation of Nrg4 enhanced the expression of proinflammatory cytokines. Anti-inflammatory agents recovered the insulin receptor, but not Glut4, content. Proteins enriched in Glut4 storage vesicles such as the insulin-responsive aminopeptidase (IRAP) and Syntaxin-6 as well as TBC1D4, a protein involved in the intracellular retention of Glut4 vesicles, also decreased by Nrg4 KD. Insulin failed to reduce autophagy in Nrg4 KD adipocytes, observed by a minor effect on mTOR phosphorylation, at the time that proteins involved in autophagy such as LC3-II, Rab11, and Clathrin were markedly upregulated. The lysosomal activity inhibitor bafilomycin A1 restored Glut4, IRAP, Syntaxin-6, and TBC1D4 content to those found in control adipocytes. Our study reveals that Nrg4 preserves the insulin responsiveness by preventing inflammation and, in turn, benefits the insulin regulation of autophagy.

## 1. Introduction

Neuregulins belong to the epidermal growth factor (EGF) family members, which are characterized by having a bioactive EGF-like domain that binds to tyrosine kinase ErbB receptors (reviewed in [1]). The neuregulin subfamily comprises at least four different genes, *Nrg1-4*, with *Nrg1* gene products being the most widely studied with regard to its strong involvement in the development and function of the nervous system, heart, and skeletal muscle [2,3,4,5,6]. Recently, Nrg4 has been postulated as an adipokine with endocrine effects whose expression in adipocytes is reduced under conditions of insulin resistance such as those related to obesity [7]. Nrg4 is a small protein with a transmembrane domain and a short extracellular portion containing the EGF-like domain, flanked at the C-terminal by a proteolytic site that upon cleavage releases the EGF domain, which then binds specifically to ErbB4 receptors [8]. *Nrg4* is mostly expressed in brown adipose tissue (BAT) and white adipose tissue (WAT), although low levels have been detected in another insulin-sensitive tissue, the liver, but no evident expression was detected in skeletal muscle or heart [7,9]. *Nrg4* in WAT is upregulated upon cold exposure, which promotes innervation and a browning phenotype [9]. Accordingly, a positive correlation has been found between *NRG4* and thermogenic/beige-related gene expression in human WAT [10]. Studies of loss- and gain-of-function of *Nrg4* or *ErbB4* in mice indicate that Nrg4 promotes a healthy phenotype. Indeed, *Nrg4* transgenic mice have elevated whole body energy expenditure and glucose utilization [11]. In liver, Nrg4 protects against the deleterious effects of a high-fat diet (HFD) by inhibiting de novo lipogenesis [7] and activating fatty acid oxidation and ketogenesis [11]. Additionally, Nrg4 downregulates the expression of proinflammatory cytokines in WAT [11] and liver [12] and induces angiogenesis in adipose tissue [13,14]. Moreover, *ErbB4* knockout mice submitted to fat diet develop obesity and insulin resistance [15]. Overall, these data suggest that the Nrg4/ErbB4 axis has a protective role upon nutritional challenge that drives insulin resistance, although the mechanisms that underlie these effects are largely obscure. Here, we focused the studies on the role of Nrg4 to preserve insulin responsiveness specifically in adipocytes. To this end, we generated and characterized a Nrg4 KD 3T3-L1 stable cell line to analyze whether the insulin action is affected and what are the mechanisms involved in these possible alterations.

## 2. Results

### 2.1. Nrg4 Deficiency Has No Effect on Differentiation of 3T3-L1 PRE-Adipocytes

**Figure 1:**
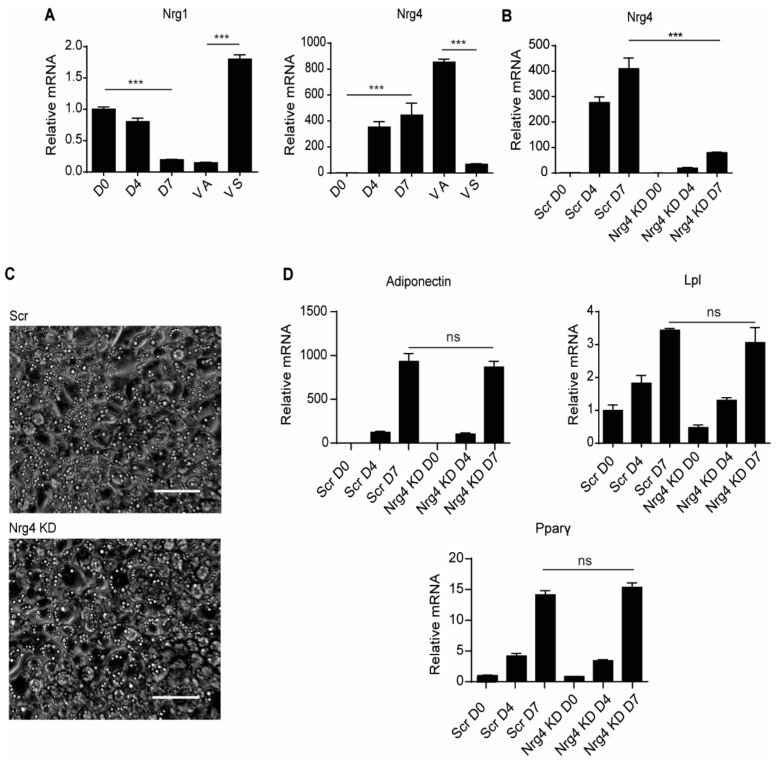
Generation of Nrg4 knockdown 3T3-L1 adipocytes. (**A**) Expression of Nrg1 and Nrg4 in 3T3-L1 pre-adipocytes at day 0 (D0), and at D4 and D7 of differentiation, and in adipocyte (VA) and stromal (VS) fractions from murine visceral white adipose tissue. Data were normalized to D0 3T3-L1 pre-adipocyte expression (*n* = 3). (**B**) Expression of Nrg4 in scramble (Scr) and Nrg4 knockdown (KD) cells at D0, D4, and D7 (*n* = 6). (**C**) Representative micrographs (20x objective) of the generated Scr and Nrg4 KD adipocytes at D7 of differentiation. Scale bar, 50 μm. (**D**) Expression of the adipogenic markers Adiponectin, Lipoprotein lipase (Lpl), and Peroxisome proliferator-activated receptor-γ (Pparγ) in Scr and Nrg4 KD cells at D0, D4, and D7 (*n* = 6). All qPCR data were normalized to Scr D0 pre-adipocyte expression. Data represent mean ± SEM. *** *p* ≤ 0.001, ns (not significant).

We first surveyed the gene expression of neuregulins in differentiating 3T3-L1 cells. We found that *Nrg1* expression decreased significantly along differentiation, reaching a similar level to that found in the mouse WAT adipocyte fraction after seven days (Figure 1A). In contrast, the WAT stromal fraction showed high expression of *Nrg1*. Analysis of *Nrg4* expression revealed the opposite behavior, with an increase in its expression during 3T3-L1 cell differentiation (Figure 1A). Notably, the WAT stromal fraction had negligible *Nrg4* expression. 3T3-L1 preadipocytes were transduced with lentiviral vectors containing a specific shRNA for *Nrg4* or scramble sequences with no effects on *Nrg4* expression. To validate the Nrg4 knockdown (Nrg4 KD) cell model, the *Nrg4* expression was analyzed in comparison with the control group (Scr) (Figure 1B). Downregulation of *Nrg4* did not affect the differentiation of 3T3-L1 cells, as evaluated by their morphological appearance (Figure 1C) and the mRNA content of several adipocyte markers including adiponectin, lipoprotein lipase (Lpl), and peroxisome proliferator-activated receptor γ (Pparγ) (Figure 1D).

### 2.2. Nrg4-Deficient Adipocytes Have Impaired Insulin-Stimulated Glucose Transport

**Figure 2:**
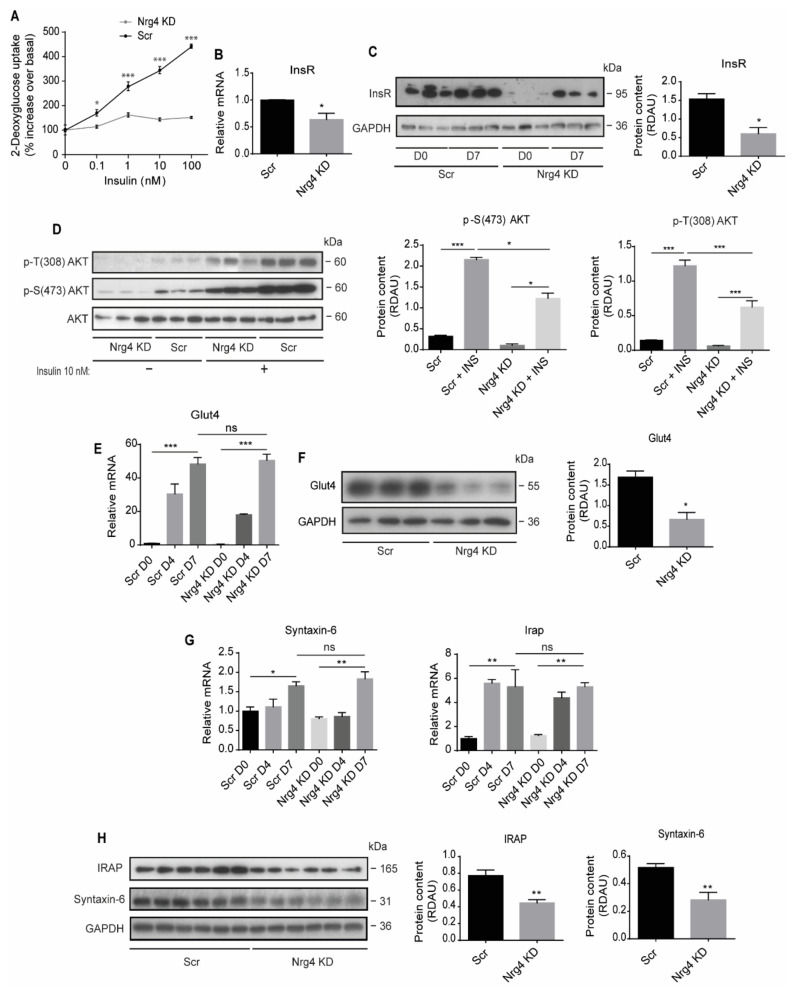
Nrg4 knockdown adipocytes are insulin resistant and show diminished insulin receptor expression and Glut4 protein content. (**A**) Concentration-response curve of the effect of insulin on 2-d-deoxyglucose uptake in Scr and Nrg4 KD adipocytes at day 7 (D7) of differentiation (*n* = 3). Cells were starved without fetal bovine serum for 16 h and then incubated with different insulin concentrations for 30 min. 2-d-deoxyglucose uptake data were expressed as the % increase relative to basal values, in nmols/mg protein × 5 min. (**B**) Insulin receptor (InsR) expression in D7 Scr and Nrg4 KD adipocytes (*n* = 3). Data were normalized to Scr adipocytes D7 expression. (**C**) Western blot bands of InsR β subunit in Scr and Nrg4 KD cells at D0 and D7 (*n* = 3). The lower band of the InsR β subunit blot at D7 was quantified. (**D**) Western blot bands and quantification of phospho (p)-T(308)AKT and p-S(473)AKT in insulin-treated and non-treated Scr and Nrg4 KD adipocytes at D7 (*n* = 3). Data were normalized to AKT protein content. (**E**) Glut4 expression in Scr and Nrg4 KD cells at D0, D4 and D7 (*n* = 6). Data were normalized to Scr D0 preadipocyte expression. (**F**) Western blot bands and quantification for Glut4 in Scr and Nrg4 KD cells at D7 (*n* = 3). (**G**) Syntaxin-6 and Insulin-regulated aminopeptidase (Irap) expression in Scr and Nrg4 KD cells at D0, D4 and D7 (*n* = 3). Data were normalized to Scr D0 preadipocyte expression. (**H**) Western blot bands and quantification of IRAP and Syntaxin-6 in Scr and Nrg4 KD cells at D7 (*n* = 6). Each lane in the blots represents an independent experiment. Quantification was normalized by the loading control, GAPDH, and expressed as relative densitometric arbitrary units (RDAU). Overall contrast and brightness of the western blot was adjusted to clarify the images. Data represent mean ± SEM. * *p* ≤ 0.05, ** *p* ≤ 0.01, *** *p* ≤ 0.001, ns (not significant).

We next studied insulin responsiveness by examining the insulin-dependent uptake of the non-metabolizable glucose analogue 2-d-deoxyglucose (2DG) in differentiated Scr and Nrg4 KD 3T3-L1 cells. As expected, 2DG uptake in Scr cells increased in an insulin concentration-dependent manner (Figure 2A). In contrast, insulin action on 2DG uptake was completely blocked in differentiated Nrg4 KD 3T3-L1 cells (Figure 2A). Analysis of insulin receptor (InsR) expression indicated that both transcript (Figure 2B) and protein (Figure 2C) levels were significantly lower in differentiated Nrg4 KD cells than in Scr cells. Likewise, the level of AKT/PKB phosphorylation upon insulin stimulation was also reduced in Nrg4 KD adipocytes (Figure 2D). Besides InsR, we found that the content in Glut4 mRNA remained unchanged between Nrg4 KD and Scr adipocytes (Figure 2E). However, there was a marked reduction in Glut4 protein in the former (Figure 2F). Previous studies in 3T3-L1 adipocytes showed that, under basal conditions, Glut4 is retained intracellularly in specialized translocation-competent compartments, termed Glut4 storage vesicles (GSVs), which are enriched in proteins including insulin-regulated aminopeptidase (IRAP) and Syntaxin-6 [16,17].

Examination of these markers showed that the mRNA levels were unaltered between differentiating Nrg4 KD and Scr adipocytes (Figure 2G), however, the protein content was significantly lower in Nrg4 KD adipocytes (Figure 2H). These results suggest that the GSV cell content is compromised in Nrg4 KD adipocytes.

### 2.3. Nrg4 KD Adipocytes Show Enhanced Inflammation

**Figure 3:**
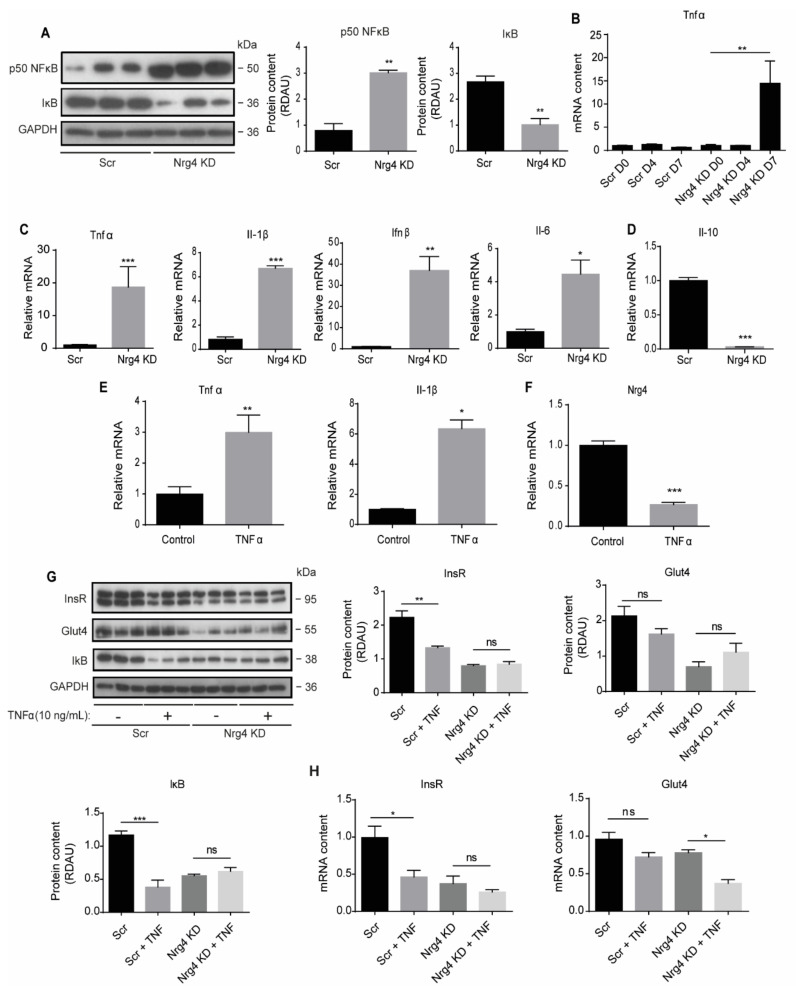
Cell-autonomous inflammation in Nrg4 knockdown adipocytes. (**A**) Western blot bands and quantification of p50 NF-κB and IκB, relative to GAPDH, in Scr and Nrg4 KD adipocytes at day 7 (D7) of differentiation (*n* = 3). Each lane represents an independent experiment. (**B**) Tnfα expression during differentiation in Scr and Nrg4 KD cells (*n* = 3). Data were normalized to Scr D0 preadipocyte expression. (**C**) Expression of the NF-κB target genes Tnfα, Il-1β, Ifnβ, Il-6, and (**D**) expression of the anti-inflammatory cytokine Il-10 in Scr and Nrg4 KD adipocytes at D7 (*n* = 3). Data were normalized to Scr D7 expression. (**E**,**F**) Tnfα, Il-1β, and Nrg4 expression in TNFα-treated and non-treated (Control) 3T3-L1 adipocytes at D7 (*n* = 3). Adipocytes were treated with 10 ng/mL TNFα for 24 h at D6. Data were normalized to non-treated control adipocyte expression at D7. (**G**) Western blot bands and quantification of InsR, Glut4, and IκB in TNFα-treated and non-treated Scr and Nrg4 KD adipocytes at D7 (*n* = 3). The lower band of the InsR blot was quantified. Overall contrast and brightness of the western blots were adjusted to clarify the images. (**H**) InsR and Glut4 expression in TNFα-treated and non-treated Scr and Nrg4 KD adipocytes at D7 (*n* = 3). Data were normalized to non-treated Scr adipocytes. Data represent mean ± SEM. * *p* ≤ 0.05, ** *p* ≤ 0.01, *** *p* ≤ 0.001, ns (not significant).

Insulin resistance is known to be associated with chronic low-grade inflammation in WAT, which requires the activation of the transcription factor NF-κB [18]. Hence, we next examined the protein content of NF-κB and its natural inhibitor IκB in differentiated adipocytes. Expression of the NF-κB p50 subunit was significantly greater in Nrg4 KD than in Scr adipocytes (Figure 3A). Consistent with this finding, the expression of proinflammatory cytokines including Tnfα (Tumor necrosis factor α), Il-1β (Interleukin-1β), Il-6, and Ifnβ (Interferon β) was significantly higher in Nrg4 KD than in Scr adipocytes (Figure 3C), whereas the expression of Il-10, an anti-inflammatory cytokine, was significantly lower (Figure 3D). It has been previously reported that TNFα suppresses adipogenesis [18]. However, we observed that the increase in Tnfα expression occurred late during adipogenesis in Nrg4 KD cells, when they were already fully differentiated (Figure 3B). Treatment of Scr cells with TNFα (10 ng/mL) during the last 24 h of differentiation increased the inflammatory profile, as observed by the increase in the expression of Tnfα and Il-1β (Figure 3E). As previously described [7,11], exogenous administration of TNFα reduced Nrg4 expression in Scr adipocytes (Figure 3F). Addition of TNFα reduced the protein content of IκB in Scr cells but did not further decrease in Nrg4 KD adipocytes (Figure 3G). Similarly, addition of TNFα significantly reduced the mRNA and protein content of the InsR in Scr cells but did not cause a further decrease in Nrg4 KD cells (Figure 3G,H). Analysis of the GLUT4 expression showed that the addition of TNFα to Scr cells caused a small but non-significant decrease in mRNA and protein levels. However, the addition of TNFα to Nrg4 KD adipocytes significantly decreased Glut4 mRNA but had no further impact at the protein level (Figure 3G,H). Overall, these results suggest a negative relationship between the expression of Tnfα and InsR in Nrg4 KD adipocytes. In contrast, the loss of Glut4 protein observed in Nrg4 KD adipocytes did not seem to be associated with the increase in Tnfα expression observed in these cells.

### 2.4. Treatment of Nrg4 KD Adipocytes with Conditioned Medium from Control Adipocytes or with Recombinant Nrg4 Reverses Inflammation and Restores Insulin Receptor and Glut4 Content

**Figure 4:**
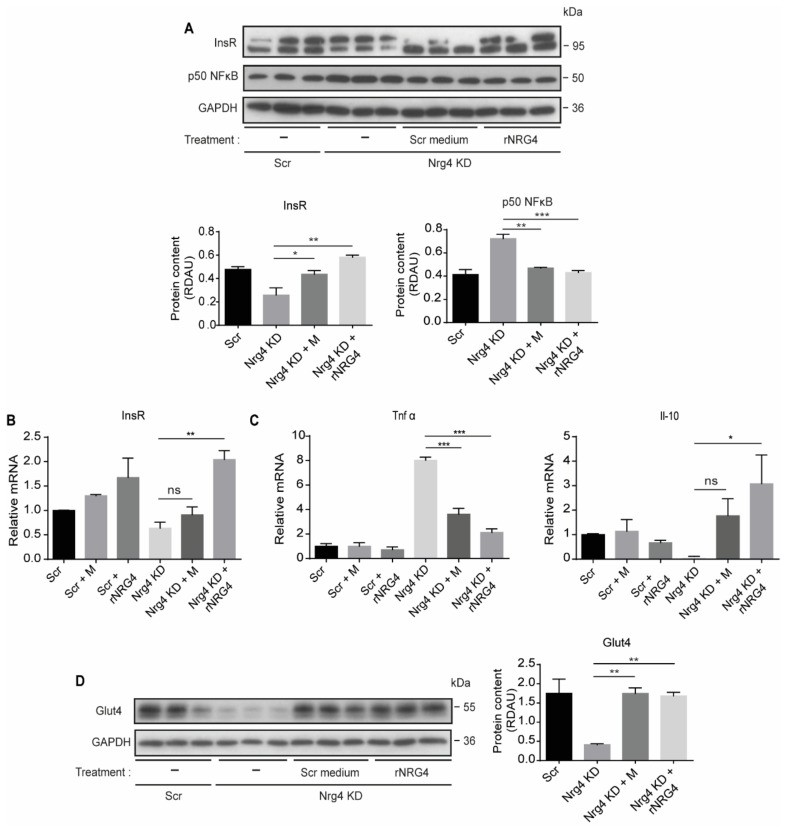
Human recombinant Nrg4 and Scr conditioned medium treatment of Nrg4 KD adipocytes restores Glut4 and InsR protein content and reverses cell-autonomous inflammation. Adipocytes were treated with 2 mL of conditioned medium from Scr D7 adipocytes, or with 50 ng/mL rNRG4 at D5 for 48 h. (**A**) Western blot bands and quantification of p50 NF-κB and InsR proteins in non-treated Scr adipocytes, and in non-treated, Scr conditioned medium (M)- or recombinant Nrg4 (rNRG4)-treated Nrg4 KD adipocytes at day 7 (D7) of differentiation (*n* = 3). The lower band of the InsR blot was quantified. (**B**) InsR expression in non-treated, Scr M or rNRG4-treated Scr, and Nrg4 KD adipocytes at D7 (*n* = 3). (**C**) Tnfα and Il-10 expression in non-treated, Scr M, or rNRG4-treated Scr and Nrg4 KD adipocytes at D7 (*n* = 3). All the qPCR data were normalized to non-treated Scr adipocyte expression at D7. (**D**) Western blot bands and quantification of Glut4 in non-treated Scr adipocytes, and non-treated, Scr M-, and rNRG4-treated Nrg4 KD adipocytes at D7 (*n* = 3). In the western blots, GAPDH was used as a loading control. Overall contrast and brightness of the western blot were adjusted to clarify the images. Data represent mean ± SEM. * *p* ≤ 0.05, ** *p* ≤ 0.01, *** *p* ≤ 0.001, ns (not significant).

To validate previous data, we supplemented Nrg4 KD adipocyte cultures with Nrg4. Specifically, we treated Nrg4 KD adipocytes with conditioned medium from differentiated Scr 3T3-L1 adipocytes, which contains endogenously secreted Nrg4, and with 50 ng/mL bioactive human recombinant Nrg4, a concentration that is within the range reported for Nrg4 in mouse serum [19]. Both treatments decreased the content of NF-κB to the levels observed in control Scr cells (Figure 4A), reducing the Tnfα expression and increasing the Il-10 expression (Figure 4C). Under these conditions, the expression of InsR was restored and the protein content of both InsR and Glut4 was normalized to the Scr cells levels (Figure 4A,B,D).

### 2.5. Sodium Salicylate and Dexamethasone Restore the Expression of the Insulin Receptor, but Not the Content of Glut4 Protein in Nrg4 KD Adipocytes

**Figure 5:**
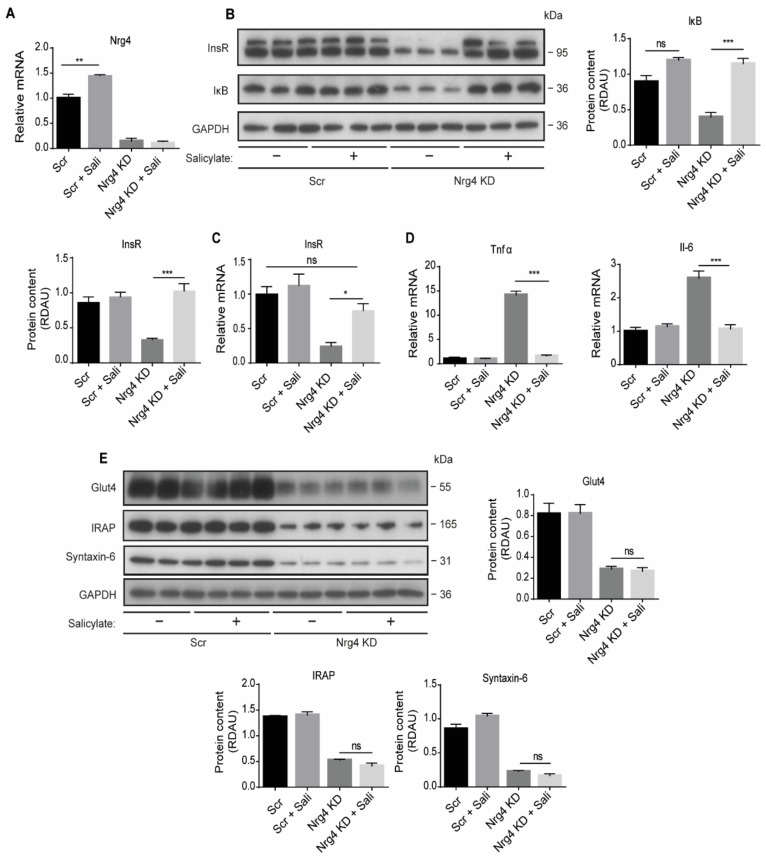
Salicylate treatment reverses the cell-autonomous inflammatory phenotype upon Nrg4 loss-of-function: Adipocytes were treated with 1 mM salicylate (Sali) at D6 for 24 h. (**A**) Nrg4 expression in Sali-treated or non-treated Scr and Nrg4 KD adipocytes at day 7 (D7) of differentiation (*n* = 3). (**B**) Western blot bands and quantification of InsR β subunit (quantification of the lower band) and IκB protein in Sali-treated or non-treated Scr and Nrg4 KD adipocytes at D7 (*n* = 3). (**C**) InsR expression in Sali-treated or non-treated Scr and Nrg4 KD adipocytes at D7 (*n* = 3). (**D**) Tnfα and Il-6 expression in Sali-treated or non-treated Scr and Nrg4 KD adipocytes at D7 (*n* = 3). All the qPCR data were normalized to non-treated Scr adipocyte expression at D7. (**E**) Western blot bands and quantification of Glut4, IRAP and Syntaxin-6 protein in Sali-treated or non-treated Scr and Nrg4 KD adipocytes at D7 (*n* = 3). In the western blots, GAPDH was used as a loading control. Each lane of the blots represents an independent experiment. Overall contrast and brightness of the western blots were adjusted to clarify the images. Data represent mean ± SEM. * *p* ≤ 0.05, ** *p* ≤ 0.01, *** *p* ≤ 0.001, ns (not significant).

To discern which alterations were directly caused by inflammation, we treated Nrg4 KD adipocytes with the anti-inflammatory agent sodium salicylate. Salicylate administration increased Nrg4 expression in Scr cells, but not in Nrg4 KD cells (Figure 5A). Salicylate treatment of Nrg4 KD adipocytes completely restored the protein content of IκB (Figure 5B). This increase in IκB was concomitant with a reduction in the expression of the Tnfα and Il-6 (Figure 5D). Salicylate also re-established the expression of InsR in Nrg4 KD adipocytes (Figure 5B,C) but was unable to restore the protein content of Glut4, IRAP, and Syntaxin-6 (Figure 5E). Similar results were obtained with the anti-inflammatory factor dexamethasone, used at a subadipogenic concentration (200 nM) (Figure S1), confirming the results obtained with salicylate.

### 2.6. Autophagy Is Enhanced in Nrg4 KD Adipocytes

**Figure 6:**
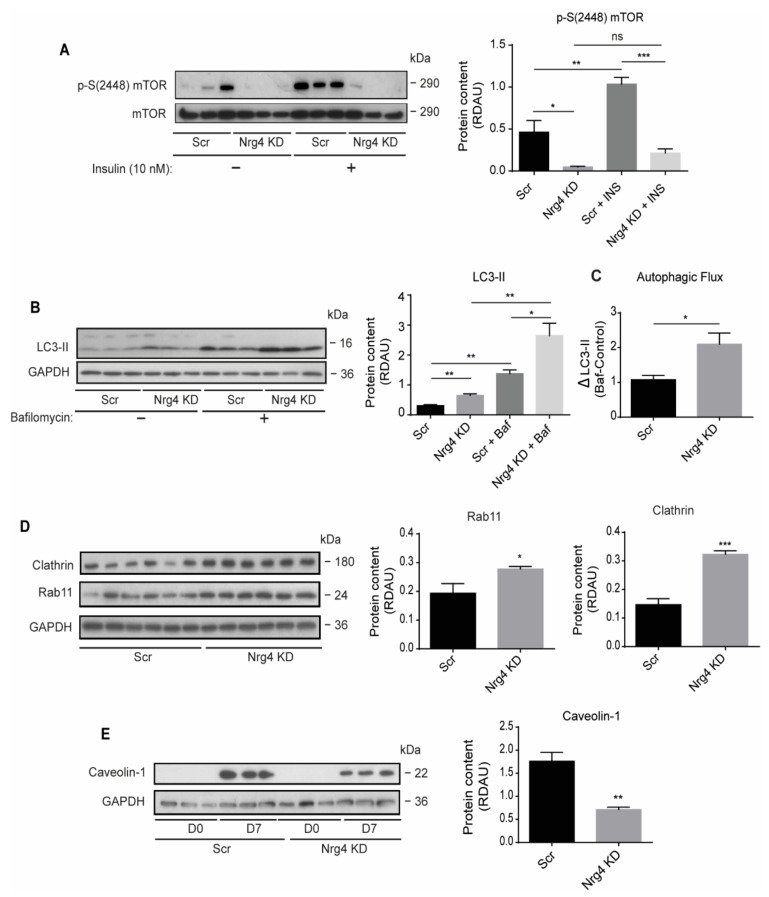
Nrg4 knockdown adipocytes show accelerated autophagy flux and deficient mTORc1 regulation upon insulin treatment: (**A**) Cells were starved without fetal bovine serum for 16 h and then incubated with 10 nM insulin for 30 min. Western blot bands and quantification of phospho (p)-S(2448) mTOR in non-treated and insulin-treated Scr and Nrg4 KD adipocytes at day 7 (D7) of differentiation (*n* = 3). P-S(2448) mTOR protein content was normalized to total mTOR protein content. (**B**) Adipocytes were treated with 200 nM bafilomycin A1 for 2 h. Western blot bands and quantification of LC3-II in non-treated or bafilomycin A1-treated Scr and Nrg4 KD adipocytes at D7 (*n* = 3). (**C**) Autophagy flux determination in Scr and Nrg4 KD adipocytes. ΔLC3-II values were calculated by subtracting the mean of LC3-II RDAU values from the non-treated cells to the bafilomycin-treated LC3-II RDAU values (*n* = 3). (**D**) Western blot bands and quantification of Clathrin and Rab11 in Scr and Nrg4 KD adipocytes at D7 (*n* = 6). Western blots from panel 6D and 2H were performed simultaneously with a single control experiment. (**E**) Representative western blot of Caveolin-1 in Scr and Nrg4 KD adipocytes at D0 and D7 of differentiation, and quantification at D7 (*n* = 3). Western blots from panels 6E and 2C were performed simultaneously with a common loading control, GAPDH. Each lane of the blots represents an independent experiment. Overall contrast and brightness of the western blots were adjusted to clarify the images. Data represent mean ± SEM. * *p* ≤ 0.05, ** *p* ≤ 0.01, *** *p* ≤ 0.001, ns (not significant). For the statistical analysis of the graph in panel B, *t*-tests was used to analyze the statistical differences between groups.

High nutrient availability and insulin activate the mechanistic target of rapamycin (mTOR) complex 1 (mTORc1), which regulates cell growth and metabolism and represses autophagy (reviewed in [20]). Our findings of reduced GSV proteins content in Nrg4 KD adipocytes suggest that these Glut4 vesicles might be degraded by autophagy. So, we monitored the activity of mTORc1 [21] by determining mTOR phosphorylation at a conserved serine residue (S2448) [22]. Of note, basal mTOR phosphorylation was significantly lower in Nrg4 KD than in Scr adipocytes, suggesting diminished mTORc1 activity (Figure 6A). Moreover, whereas insulin induced mTOR phosphorylation in Scr adipocytes, no significant effects were evident in Nrg4 KD cells, supporting the hypothesis that autophagy was not properly inhibited in response to insulin in Nrg4-deficient adipocytes (Figure 6A). To confirm these findings, we analyzed the content of the microtubule-associated protein light chain 3B form II (LC3-II), a marker of autophagosomes [23]. LC3-II was significantly increased in Nrg4 KD adipocytes, both under basal conditions and upon treatment with bafilomycin A1, a disruptor of lysosomal activity (Figure 6B). These results point to the existence of a greater amount of autophagosomes in Nrg4 KD adipocytes and, consequently, a higher autophagic flux. The absolute increase in LC3-II upon bafilomycin A1 treatment was significantly higher in Nrg4 KD cells (Figure 6C), confirming that they show a higher autophagic flux. We also analyzed the protein content of Rab11 and Clathrin, which have essential roles in the formation of autophagosomes [24] and autolysosomes [25], respectively. The protein content of both markers was higher in Nrg4 KD adipocytes (Figure 6D). Deficiency in the expression of Caveolin-1 also induces autophagy [26,27,28]. We observed that the cellular content in Caveolin-1 was significantly lower in Nrg4 KD than in Scr adipocytes (Figure 6E). Hence, the results indicate that the control of autophagy is, at least partly, disabled in Nrg4 KD adipocytes.

### 2.7. Blockade of Autophagy Restores Glut4 and GSV Proteins Content in Nrg4 KD Adipocytes

**Figure 7:**
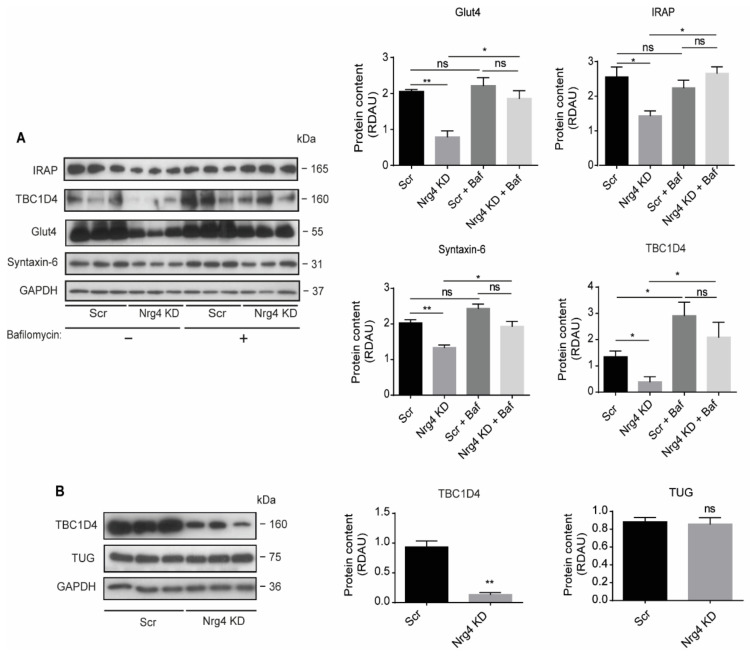
Depletion of Glut4 and GSV-associated proteins in Nrg4 knockdown adipocytes is caused by autophagy degradation. (**A**) Adipocytes were treated with 200 nM bafilomycin A1 for 2 h. Western blot bands and quantification of IRAP, TBC1D4, Glut4, and Syntaxin-6 proteins in non-treated and bafilomycin A1-treated Scr and Nrg4 KD adipocytes at day 7 (D7) of differentiation (*n* = 3). (**B**) Representative western blot and quantification of TBC1D4 and TUG in Scr and Nrg4 KD adipocytes at D7 (*n* = 3); GAPDH was used as a loading control. Each lane of the blots represents an independent experiment. Overall contrast and brightness of the whole western blots were adjusted to clarify the images. Data represent mean ± SEM. * *p* ≤ 0.05, ** *p* ≤ 0.01, ns (not significant). For the statistical analysis of the graph in panel 7D graph, *t*-tests were used to analyze the statistical differences between groups.

To analyze whether the GSV are degraded through autophagy, we determined the Glut4 content in the absence or presence of bafilomycin A1. Bafilomycin A1 treatment for 2 h did not change Glut4 protein content in Scr cells, but it completely recovered the Glut4 content in Nrg4 KD adipocytes (Figure 7A). Similar results were obtained by analyzing other GSV proteins, IRAP, and Syntaxin-6 (Figure 7A). These results strongly suggest that GSV are degraded in Nrg4 KD through autophagy. Finally, we examined the expression of TBC1 domain family member 4 (TBC1D4, also known as AS160), a Rab GTPase activating protein involved in the intracellular retention of GSV under basal conditions. Upon insulin action, AKT phosphorylates TBC1D4, which detaches from GSV and allows their translocation to the plasma membrane [29,30,31,32]. We observed a profound loss in the protein content of TBC1D4 in Nrg4 KD adipocytes (Figure 7B), without changes in the Tether containing a UBX domain for Glut4 (TUG) protein, which some authors involved in the intracellular retention of GSV in adipocytes [33]. TBC1D4 protein content was completely restored in Nrg4 KD adipocytes by bafilomycin A1 (Figure 7A).

## 3. Discussion

We show that Nrg4 KD does not affect adipogenesis but has a deleterious effect on insulin responsiveness in 3T3-L1 adipocytes, increasing the expression of inflammatory cytokines and enhancing autophagy.

We observed that adipogenesis is accompanied by a switch in neuregulin isoform expression, with an exchange of Nrg1 for Nrg4. The high expression of Nrg1 in undifferentiated 3T3-L1 cells confirms a previous report [34] and points to Nrg1 involvement in the early events of adipogenesis as occurs in myogenesis [35]. In addition, no effect on adipogenesis has been attributed to Nrg4 yet [15], which was confirmed here by the late expression of Nrg4 during adipogenesis, the lack of impact on the expression of differentiation markers and by the presence of lipid droplets in Nrg4 KD adipocytes. In contrast, Nrg4 is essential to sustain the cellular content of the insulin receptor and Glut4 glucose transporter in 3T3-L1 adipocytes. Thus, here, we show for the first time that the downregulation of Nrg4 expression triggers insulin resistance in adipocytes. We therefore focused our attention on the mechanisms through which Nrg4 KD drives insulin resistance.

Obesity is recognized as an insulin-resistant state characterized by low-grade chronic inflammation. In this scenario, Nrg4 expression is markedly downregulated in the adipose tissue of mice and humans [7]. Moreover, Nrg4 knockout mice show an accentuated obesity-related phenotype with a high expression of proinflammatory adipokines in WAT [11] and liver [12]. Likewise, it has been reported that TNFα or IL-1β treatment can downregulate Nrg4 expression in 3T3-L1 differentiated adipocytes [7,11], which is in accordance with our present findings. Additionally, here we show that Nrg4 can temper the expression of proinflammatory cytokines in Nrg4 KD adipocytes, likely by diminishing the content and activity of the proinflammatory transcription factor NF-κB, which is also in agreement with the studies in mice. These findings allow us to propose the existence of a negative feedback loop involving Nrg4 and NF-κB. Thus, adipocytes, hepatocytes, and likely other cell types, appear to have an autonomous mechanism to repress the inflammation governed by Nrg4. Indeed, previous studies on inflammatory bowel diseases in humans and mice [36,37,38] support this view. Treatment with Nrg4 suppresses apoptosis in colonic epithelial cells induced by proinflammatory cytokines. At the same time, Nrg4 promotes apoptosis in proinflammatory tissue macrophages, limiting colonic inflammation, suggesting the existence of local effects of Nrg4 to protect against inflammation. This is likely not the only mechanism by which Nrg4 regulates inflammation. For instance, it was reported that Nrg4 enhances angiogenesis in adipose tissue [13,14], preventing hypoxia and inflammation in obesity. Indeed, transgenic expression of Nrg4 in mice was found to stimulate the expression of vascular endothelial growth factor (VEGF) [11]. Nrg4 thus emerges as a protective factor against inflammation in multiple tissues that likely benefits insulin responsiveness.

Proinflammatory cytokines can cause insulin resistance by inhibiting the insulin-signaling pathway and reducing the content of Glut4 [39,40,41]. However, the profound impact of Nrg4 KD on the content of Glut4 in adipocytes was not the result of inflammation per se since the anti-inflammatory agents, sodium salicylate, and dexamethasone failed to reverse this action but did prevent the loss of InsR expression. Rather, our data supports the notion that the mechanisms controlling autophagy are not adequately operating in Nrg4 KD adipocytes and, likely, this is the cause of the reduction in Glut4 content. This concept is supported by our findings that Nrg4 KD adipocytes are unable to maintain basal mTORc1 activity and to sustain the insulin action on mTORc1 that should drive inhibition of autophagy. This would be in accord with the increase in the protein content of Rab11, a GTPase involved in autophagosome formation (reviewed in [20,42]) that is under the control of protein complexes that regulate mTORc1 activity [24]. Finally, the increase in LC3-II, which specifically accounts for the cell content in autophagosomes, confirms that autophagy is enhanced in Nrg4 KD adipocytes.

Our data support the view that the unique feature that favors this dysfunctional autophagy response is the downregulation of Nrg4 expression and, accordingly, Nrg4 likely plays an essential role in repressing autophagy. Further research has to explore the mechanisms by which Nrg4 regulates autophagy, beyond its protective role on insulin sensitivity. The fact that inflammation occurs late during adipogenesis in Nrg4 KD cells suggests that it does not precede autophagy. In this context, the enhanced autophagy flux observed in Nrg4 KD adipocytes affects the Glut4-enriched endosomal compartment. GSV moves at a continuous low rate between the intracellular location and the surface membrane in non-insulin-stimulated conditions. GSV recycles to recover intracellular pools by the generation of early endosomes through clathrin-coated pits or caveolaes [43]. Clathrin and Caveolin-1, both highly present in the surface membranes of adipocytes, play different roles in the newly formed endosomal Glut4 vesicles returning to the storage pool. Clathrin participates in the transport from endosomes to the Golgi apparatus [44] but, as previously mentioned, it is also involved in the formation of autolysosomes [25]. In contrast, Caveolin-1 is required to maintain Glut4 content in 3T3-L1 adipocytes, since its downregulation leads to higher Glut4 levels in the lysosomal compartment [45]. Here, we show that in Nrg4 KD, the cellular content in Clathrin increases significantly, whereas the Caveolin-1 content decreases, suggesting that a decrease in the Cavelin-1/Clathrin ratio may signal GSV for autophagosome engulfment. TBC1D4 binds to IRAP in GSV and has been proposed to be responsible for the intracellular retention of Glut4 vesicles under basal conditions. Following the action of insulin, the phosphorylation of TBC1D4 by AKT would inhibit TBC1D4 binding to IRAP, resulting in the release of GSV to the plasma membrane (reviewed in [32]). The loss of TBC1D4 observed in Nrg4 KD adipocytes could be a consequence of the higher autophagy flux, since the content of TBC1D4 is recovered by bafilomycin A1 treatment. We thus suggest that TBC1D4 could be degraded by autophagy. If so, additional studies have to address the mechanism by which TBC1D4 may be trapped in the autophagy pathway.

In summary, Nrg4 appears to play an essential role preserving insulin responsiveness by protecting against inflammation and exacerbated autophagy in adipocytes. Future studies will have to delineate the Nrg4-induced mechanisms that allow to protect adipocytes from inflammation and deleterious autophagy in order to prevent insulin resistance.

## 4. Materials and Methods

### 4.1. Reagents

High-glucose (4.5 g/L) containing Dulbecco’s modified Eagle’s medium (DMEM #L0104-500) was purchased from Biowest (Nuaillé, France). Calf serum (#16170078), fetal bovine serum (FBS) (#10270106), penicillin/streptomycin (#15140122), and puromycin (#A1113803) were all purchased from Gibco (Tavarnuzze, Italy). *Eschericia coli* containing the pLKO.1-Puro plasmid and expressing a short-hairpin interfering RNA (shRNA) against *Nrg4* (GCCTGGTAGAGACAAACAATA) or a scrambled (Scr) control (SHC002) were obtained from the Functional Genomics facility of the Institute for Research in Biomedicine (IRB) (Barcelona, Spain). The PureLink™ HiPure Plasmid DNA Kit (#K210005), RNA Mini Kit columns (#12183018A), deoxynucleotides (#R0181), oligodTs (#18418020), RNAseOUT™ solution (#10777019), and SuperScript™ II reverse transcriptase (#18064014) were all purchased from Invitrogen (Waltham, MA, USA). SYBR™ Green PCR Master Mix (#4367659) was obtained from Applied Biosystems (Waltham, MA, USA). Polyethylenimine-2500 (PEI-2500) (#24313) was obtained from Polysciences (Warrington, PA, USA). TNFα (#300-01A) was obtained from Peprotech (London, UK). Human recombinant neuregulin 4 (#RKQ8WWG) was purchased from Reprokine (Rehovot, Israel). Bafilomycin A1 (#sc-201550) was purchased from Santa Cruz Biotechnology (Dallas, TX, USA). Protease inhibitor cocktail (#78430) and the Pierce™ Bicinchoninic Acid Protein Assay Kit (BCA) (#23225) were purchased from Thermo Scientific (Waltham, MA, USA). Phosphatase inhibitor (#04906845001) was obtained from Roche (Basel, Switzerland) Molecular-weight size marker Hyper PAGE was obtained from Bioline (London, UK). PVDF membranes (#IPVH00010) were purchased from MERCK Millipore (Darmstadt, Germany). The anti-Glut4 polyclonal antibody, raised against the 15 C-terminal amino acid residues, OSCRX, was produced in our laboratory [46]. The polyclonal antibodies anti-insulin receptor β chain (InsR) (#611277) and anti-Caveolin-1 (#610406) were purchased from BD Transduction Laboratories (San Jose, CA, USA). The monoclonal antibodies anti-GAPDH (#5174T), anti-Clathrin (#4796), anti-Syntaxin-6 (#2869), and anti-AS160/TBC1D4 (#2670) as well as the polyclonal antibodies anti-AKT (#9272), anti-phospho-T(308)AKT (#9275), anti-phospho-S(473)AKT (#9271), anti-IRAP (#3808), anti-mTOR (#2972), anti-phospho-S(2448) mTOR (#2971), anti-TUG (#2049), anti-LC3B (#2775), and anti-Rab11a (#2413) were all purchased from Cell Signaling Technologies (Danvers, MA). The polyclonal antibodies anti-NF-κB p-50 (#sc-114) and anti-IkB-α (#sc-203) were obtained from Santa Cruz Biotechnology. Horseradish peroxidase (HRP)-conjugated anti-rabbit (#711-035-152) and anti-mouse (#715-035-150) secondary antibodies were obtained from Jackson ImmunoResearch (Soham, UK). The ECL™ Kit (#15387655) was purchased from Amersham GE Healthcare (Buckinghamshire, UK). Bovine serum albumin (BSA) (#6003), human recombinant insulin (#I5500), dexamethasone (#D2915), rosiglitazone (#R2408), 3-isobutyl-1-methylxanthine (IBMX) (#I5879), ampicillin (#A1593), Luria Bertani broth (#L3022), Polybrene^®^ transfection reagent (#TR-1003-G), sodium salicylate (#S3007), and gene-specific primers were obtained from Sigma-Aldrich (St. Louis, MO, USA). Other commonly used chemicals were purchased from Sigma-Aldrich.

### 4.2. White Adipose Tissue Fractionation

Visceral WAT (vWAT) was obtained from 6-month-old wild-type C57/BL6 mice of the Animal Facility of the Faculty of Biology, University of Barcelona. All procedures with mice were performed with the approval of the Institutional Animal Care and Use Committee of this institution. Two males and one female mice, were anesthetized with isoflurane, and vWAT was obtained from the peri-gonadal pads. Adipocytes and stromal-enriched fractions were isolated by enzymatic digestion with type 2 collagenase and centrifuged at 1600 rpm for 10 min. The supernatant, containing the adipocyte fraction was separated from the pellet, containing the stromal fraction. Finally, total RNA was extracted as described in Section 4.7.

### 4.3. T3-L1 Adipocyte Cell Culture and Differentiation

Mice embryo 3T3-L1 fibroblast (preadipocytes) cell line was originally obtained from ATCC (CL-173; American Type Culture Collection, Manassas, VA, USA). Preadipocytes were grown in 10% (*v*/*v*) activated calf serum in high-glucose DMEM with 100 U/mL of penicillin/streptomycin at 37 °C and 8% CO_2_. Adipocyte differentiation was induced by adding 1 μg/mL insulin, 0.25 μM dexamethasone, 1 μM rosiglitazone, 0.5 mM IBMX, and 10% (*v*/*v*) activated FBS to high-glucose DMEM for 72 h. Subsequently, cells were cultured in 10% (*v*/*v*) FBS high-glucose DMEM medium supplemented with 1 μg/mL insulin for 24 h. Finally, cells were incubated in non-supplemented 10% (*v*/*v*) FBS high-glucose DMEM medium for 72 h. The overall procedure lasted seven days.

### 4.4. Lentiviral Production and Infection of 3T3-L1 Preadipocytes

The PureLink™ HiPure Plasmid DNA Kit was used to obtain shRNA-pLKO.1, pCMV-dR8.2, and pMD2.G plasmids from transformed *E. coli*. Bacteria containing the pLKO.1-Puro plasmid and expressing a short-hairpin interfering RNA (shRNA) against *Nrg4* (GCCTGGTAGAGACAAACAATA) or a scrambled (Scr) control (SHC002) were obtained from the Functional Genomics facility of the Institute for Research in Biomedicine (IRB) (Barcelona, Spain). The PCMV-dR8.2 helper packaging construct and pMD2.G plasmids were provided by Dr Trono D. (EcolePolytechnique Federale de Lausanne, Switzerland). Lentiviral transduction was performed to deliver shRNAs to 3T3-L1 preadipocytes as previously described [47]. Both Scr shRNA expressing cells and Nrg4 KD preadipocytes clones were maintained in 2.5 μg/mL puromycin.

### 4.5. Cell Treatments

To analyze insulin action, fully differentiated 3T3-L1 adipocytes were preincubated for 16 h in high-glucose DMEM without serum and supplemented with 0.2% (*w*/*v*) BSA. To study insulin responsiveness in a 2-d-deoxyglucose uptake assay, a range of insulin concentrations were tested for 30 min at 37 °C (Section 2.6). Insulin signaling in adipocytes was assessed by treating cells with 10 nM insulin for 30 min at 37 °C. For studies with TNFα, adipocytes were treated with 10 ng/mL TNFα at day 6 of differentiation for 24 h. Rescue studies for *Nrg4* silenced cells were performed by incubating Nrg4 KD cells with 2 mL of conditioned medium from fully differentiated Scr 3T3-L1 adipocytes at day 5 of differentiation for 48 h. In parallel, Nrg4 KD cells were treated with 50 ng/mL human recombinant NRG4 (rNrg4) during the final 48 h of differentiation. For sodium salicylate or dexamethasone treatments, adipocytes were treated with 1 mM sodium salicylate or 200 nM dexamethasone, at day 5 of differentiation for 48 h. Finally, to assess autophagy, differentiated 3T3-L1 adipocytes were treated with 200 nM bafilomycin A1 for 2 h.

### 4.6. Measurement of 2-d-Deoxyglucose Uptake

2-d-deoxyglucose uptake was performed as described [46]. Data were normalized to protein content in cell lysates. Protein concentration was quantified with the Pierce™ BCA Protein Assay Kit.

### 4.7. RNA Extraction and Quantitative PCR

Total RNA was extracted from 3T3-L1 and from WAT, adipocytes, and stromal fractions using PureLink™ RNA Mini Kit columns. RNA samples were quantified using the Nanodrop™ 2000/2000c spectrometer from Thermo Scientific (Waltham, MA, USA). RNA was reverse transcribed using SuperScript™ II reverse transcriptase. Ten ng of cDNA was used for quantitative PCR (qPCR) with SYBR^®^ Green PCR Master Mix on the ABI Prism 7900 HT qPCR platform using SDS software of Applied Biosystems (Waltham, MA, USA) from the Genomics Facility of the Scientific and Technological Center, University of Barcelona. The primer sequences are listed in Supplementary Table S1. Gene specific primers were obtained from Sigma-Aldrich. Gene expression measurements were normalized to the housekeeping gene acidic-ribosomal protein (*Arp*) cDNA using the 2−*^ΔΔ^C_t_* method.

### 4.8. Protein Extraction and Western Blotting

Total protein extracts were prepared from cultured 3T3-L1 cells on plates, washed twice with ice-cold PBS, and scraped into lysis buffer consisting of 20 mM Tris-HCl, pH 7.5, 150 mM NaCl, 1 mM EDTA, 1 mM EGTA, 1% (*v*/*v*) NP-40, 0.1% (*m*/*v*) sodium dodecyl sulfate, and 0.5% (*w*/*v*) sodium deoxycholate, supplemented with phosphatase and protease inhibitors cocktails. Equal amounts of protein lysates were separated by SDS-PAGE and transferred to Immobilon PVDF membranes, which were then blocked with 5% (*w*/*v*) non-fat dry milk for the detection of non-phosphorylated proteins or with 3% (*w*/*v*) BSA for phosphorylated proteins in 0.1% (*v*/*v*) Tween-20 Tris-buffered saline solution (TBST) for 1 h at room temperature. All primary antibodies were diluted in TBST containing 3% BSA (*w*/*v*). Horseradish peroxidase (HRP)-conjugated anti-rabbit (#711-035-152) and anti-mouse (#715-035-150) secondary antibodies were obtained from Jackson ImmunoResearch (Soham, UK). Secondary antibodies were diluted 1/20,000 in 5% (*w*/*v*) non-fat dry milk in TBST. ImageJ (NIH) was used to quantify blots. Relative densitometric units (RDAU) were calculated by normalizing data to the loading control GAPDH, unless stated otherwise. Overall contrast and brightness of the western blot images were adjusted to help clarify data without distorting the image.

### 4.9. Statistical Analysis

Data are presented as mean ± SEM. Comparisons between two experimental groups were analyzed using the Student’s *t*-test. Comparisons between more than two experimental groups were analyzed with one-way analysis of variance with Tukey’s honest significant difference post-hoc test. *p*-values of significance are indicated in the figure’s captions. Data were analyzed using GraphPad Prism 6 software (San Diego, CA, USA). Figures were assembled using Adobe illustrator^®^ software (Adobe Systems Inc., San Jose, CA, USA).

## Supplementary Materials

The following are available online at https://www.mdpi.com/article/10.3390/ijms222312960/s1.
