# Supplementary material for: Neuregulin 4 Downregulation Induces Insulin Resistance in 3T3-L1 Adipocytes through Inflammation and Autophagic Degradation of GLUT4 Vesicles
Source: Int J Mol Sci. 2021 Nov 30;22(23):12960. doi: 10.3390/ijms222312960 (PMC8657571; doi:10.3390/ijms222312960)
Supplement: Supplementary file 1 [file ijms-22-12960-s001.zip › Supplementary Materials - Gum¿ñ et al - reviewed.pdf]

Supplementary Materials

Neuregulin 4 downregulation induces insulin resistance in 3T3-L1 adipocytes through inflammation and autophagic degradation of GLUT4 vesicles

Francisco Díaz-Sáez [1,2], Carla Blanco-Sinfreu [1], Adrià Archilla-Ortega [1], David Sebastian [1,3,4], Montserrat Romero [1,3,4], Maria Isabel Hernández-Alvarez [1,2,3], Sílvia Mora [1,2], Xavier Testar [1,2,3], Wifredo Ricart [5,6,7], José Manuel Fernández-Real [5,6,7], José María Moreno-Navarrete [5,6,7], Julián Aragonés [8,9], Marta Camps [1,2,3], Antonio Zorzano [1,3,4,*] and Anna Gumà [1,2,3,*]

1 Department of Biochemistry and Molecular Biomedicine, Faculty of Biology, University of Barcelona, Av. Diagonal, 643, Barcelona, Spain, 08028; frandiazsaez@gmail.com (F.D.-S.); carlablancosinfreu@outlook.com (C.B.-S.); adria.archilla.ortega@gmail.com (A.A.); mihernandez@ub.edu (M.I.H.-A.); smora@ub.edu (S.M.); xtestar@ub.edu (X.T.); martacamps@ub.edu (M.C.)
2 Institute of Biomedicine of the University of Barcelona (IBUB), Barcelona , Spain, 08028
3 Centro de Investigación Biomèdica en Red Diabetes y Enfermedades Metabólicas Asociadas (CIBERDEM), Carlos III Health Institute, Madrid, Spain, 28029; david.sebastian@irbbarcelona.org (D.S.); montserrat.romero@irbbarcelona.org (M.R.)
4 Institute for Research in Biomedicine (IRB Barcelona), Barcelona Institute of Science and Technology (BIST), Barcelona, Spain, 08028
5 Department of Medicine, Universitat de Girona, Carrer Emili Grahit, 77, Girona, Spain, 17071; wricart@idibgi.org (W.R.); jmfreal@idibgi.org (J.M.F.-R.); jmoreno@idibgi.org (J.M.M.-N.)
6 Department of Diabetes, Endocrinology and Nutrition, Institut d'Investigació Biomèdica de Girona (IDIBGI), Carrer del Dr. Castany, s/n, Salt, Spain, 17190
7 Centro de Investigación Biomèdica en Red Fisiopatología de la Obesidad y Nutrición (CIBEROBN), Carlos III Health Institute, Madrid, Spain, 28029
8 Research Unit, Hospital of Santa Cristina, Research Institute Princesa, Autonomous University of Madrid, c/Maestro Vives, 2, Madrid, Spain, 28009; jaragones.hlpr@salud.madrid.org
9 Centro de Investigación Biomédica en Red Enfermedades Cardiovasculares, Carlos III Health Institute, Madrid, Spain, 28029
* Correspondence: antonio.zorzano@irbbarcelona.org (A.Z.); aguma@ub.edu (A.G.)

Supplementary Figure S1

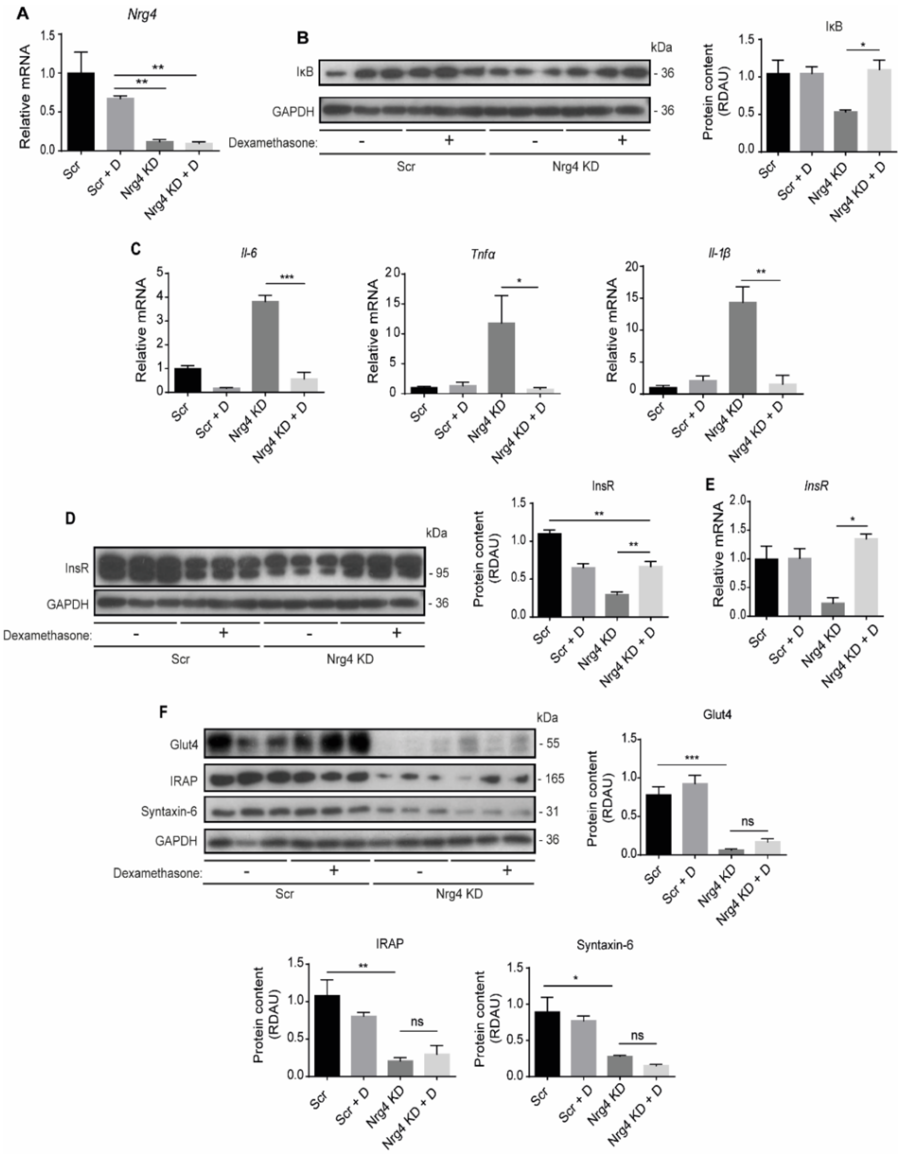

Figure S1. Dexamethasone treatment reverses the cell-autonomous inflammatory phenotype and recovers InsR protein content upon Nrg4 knockdown. Adipocytes were treated with 200 nM dexamethasone (D) at D5 for 48 h. (**A**) *Nrg4* expression in non-treated or D-treated Scr and Nrg4 KD adipocytes at day 7 (D7) of differentiation (n=3); (**B**) Western blot bands and quantification of IκB in D-treated or non-treated Scr and Nrg4 KD adipocytes at D7 (n=3); (**C**) *Tnfα*, *Il-6* and *Il-1β* expression in non-treated or D-treated Scr and Nrg4 KD adipocytes at D7 (n=3); (**D**) Western blot bands and quantification of InsR in D-treated or non-treated Scr and Nrg4 KD adipocytes at D7 (n=3). The lower band of the InsR blot was quantified. Western blots from panel B and D were performed simultaneously with a common loading control, GAPDH; (**E**) *InsR* expression in non-treated or D-treated Scr and Nrg4 KD adipocytes at D7 (n=3). All the qPCR values were normalised to those in non-treated Scr D7 adipocytes; (**F**) Western blot bands and quantification of Glut4, IRAP and Syntaxin-6 in D-treated or non-treated Scr and Nrg4 KD adipocytes at D7 (n=3). Each lane of the blots represents an independent experiment. Overall contrast and brightness of the western blots were adjusted to clarify the images. Data represent mean ± SEM. *p≤0.05, **p≤0.01, ***p≤0.001, ns (not significant).

Supplementary Table S1

Table S1. Primer sequences used for quantitative-PCR analyses.

Target gene	Forward sequence	Reverse sequence
Adiponectin	CGGCAGCACTGGCAAGTT	CCGTGATGTGGTAAGAGAAGTAGTAGA
Arp	AAGCGCGTCCTGGCATTGTCT	CCGCAGGGGCAGCAGTGGT
Glut4	GTGACTGGAACACTGGTCCTA	CCAGCCACGTTGCATTGTAG
Il-1β	GCACTACAGGCTCCGAGATGAAC	TTGTCGTTGCTTGGTTCTCCTTGT
Ifnβ	CCCTATGGAGATGACGGAGA	CCCAGTGCTGGAGAAATTGT
Il-6	TAGTCCTTCCTACCCCAATTTCC	TTGGTCCTTAGCCACTCCTTC
Il-10	GCTCTTACTGACTGGCATGAG	CGCAGCTCTAGGAGCATGTG
Irap	TTTACCAATGATCGGCTTCAGC	ICGAACCTCGGGGCTCATATT
Lpl	GGGAGTTTGGCTCCAGAGTTT	TGTGTCTTCAGGGGTCCTTAG
InsR	ATGGGCTTCGGGAGAGGAT	GGATGTCCATACCAGGGCAC
Nrg1	TTCCCATTCTGGCTTGTCTAGT	CCAGGGTCAAGGTGGGTAG
Nrg4	CACGCTGCGAAGAGGTTTTTC	CGCGATGGTAAGAGTGAGGA
Pparγ	CCAGAGCATGGTGCCTTCGCT	CAGCAACCATTGGGTCAGCTC
Syntaxin-6	ACAGGCCGTCATGCTAGATG	GGATGGCTATGGCACACCAC
Tnfα	CCCTCACACTCAGATCATCTTCT	GCTACGACGTGGGCTACAG
